# 

**Published:** 1894-01

**Authors:** 


### INDEX TO VOLUME XLVIII.

PAGE

COMMUNICATIONS.

Abscess of the Maxillary Sinus—Treatment of, by Dr. E. Lecaudey ....... ............. 9
Aconite and Iodine in Pericementitis, by W. I. Jones, D.D.S .......................... 191
Address—Abstract of, by A. E. Baldwin, M.D., D.D.S...... 125
Address, by Dr. T. M. Allen... 417
Address, by Dr. E. P. Beadles. 435
Address, by Dr. J. D. Patterson 489
Address on the Work of the Executive Committee of the World's Columbian Dental Congress, by J. Taft, D.D.S.. .................. 55
Aluminum, by Dr. Thos. P. Hinman ...................... 429
Aluminum Cast, by Dr. Harper 451
Anæsthetics—Local, by L. E. Custer, D D.S................ 527
Anæsthetizing Sensitive Dentine, by W. H. Van Deman, D.D.S ........................ 383
Anterior Fillings, by E. P. Beadles, D.D.S. .............. 478
Anterior Teeth—Fractures of, by Dr. Geo. W. Whitefield 13
Articulation of Artificial Teeth—Some Points on the, by G. Molyneaux, D.D.S..127, 273
Articulators, by Dr. H. E. Belden ........ ..................... 473
Bichloride of Mercury, by Carrie M. Stewart, D.D.S...... 8
Bite—Method of Taking the, by Dr. J. A. Frazier........ 423
Bridge-Work by A. W. Haidle, D.D.S...... ...................... 576
Boracine—Tetraborate of Soda, by Mr. Denis................. 10
Care of Children's Teeth—Ignorance and Negligence of Parents in the, by Dr. J D. Adair ....................... 426

PAGE

Care of Infants' Teeth, by Dr. W. H. Morrison ............. 487
Cavities—Varnishing, by Dr. W. G. Browne................ 487
Children—The Teeth of, by Howard Van Antwerp, D.D.S .......................... 92
Chloroform Anæsthesia without Danger, by F. H. Pritchard, M.D .............. 516
Chloroform as an Obtundent—Method of Using, by Dr. A. C. Hewitt .................. 541
Chloroform and Ether, by C. G. Darling, M.D,............ 313
Chemistry—Physiological and Therapeutics, by Dr. O. C. Farish.......................... 424
Cleft Palate Operation, by Mr. Davies-Colley ................ 302
Clinics ................................ 543
Cocaine, by Arthur R. Cushny, A.M., M.D...................... 334
Contour Filling made in the Laboratory—Some of the Merits, by Dr. T. W. Foster .............................. 479
Crown and Bridge-Work Prudence and Gutta-Percha, by Dr. Geo. V. I. Brown.. 23
Crown—An Original, by Dr. J. H. Crossland.............. 431
Demonstrations at the World's Columbian Dental Congress........ .................... 70
Dental Caries—Etiology of, by Dr. S. B. Palmer ........561 578
Dental Caries—Relation to Certain Forms of Indigestion, by E. F. Wilson, M.D...... 78
Dental Caries and Some Popular Fallacies, by Dr. Geo. J. Frederichs ................. 540
Dental Education, by Dr. I. N. Carr ......................... 443
Dental Education—Report, by Dr. L. Ottofy.................. 498

PAGE

Dental Exostosis, by Mary Linde, D.D.S. 269
Dental Legislation, by Dr. H. B. Noble 444
Dental Legislation, by Dr. A. W. Sweeney 445
Dental Protective Association—Report of, by Dr. J. N. Crouse 492, 549
Dentistry as a Specialty of Medicine, by Dr. R. A. Rush 432
Dentistry—What Relation Shall it Hold to Medicine—Discussion 54
Dentistry—Past, Present and Future, by Dr. T. J. Watson 431
Dentistry Among the Speechless, by Charlotte E. Benton, D.D.S 573
Diseases of the Palate, by Vida A. Latham, D.D.S. 6
Diseases of the Maxillary Bones and their Periosteum, by Vida A. Latham 157, 223, 261
Dyspepsia, by Hugh M. Patton, M.D 145
Early Women in Dentistry, by Lucy Hobbs Taylor 31
Education—Dental, by Dr. I. N. Carr 443
Education—Dental, Report by Dr. L. Ottofy 498
Electricity and Painless Dentistry, by L. E. Custer, B.S., D.D.S. 394
Empyema of the Maxillary Sinus, by M. H. Fletcher, M.D., D.D.S 105
Enamel—Development of, by Dr. R. R. Andrews 58
Enemies of the Human Teeth, by A. Benson, M.D., D D.S. 164
English Tube Teeth, by John Girdwood, L.D.S. 1
Ethics, Discussion of 429
Extraction of the Trigeminal Nerve, by Dr. T. W. Brophy 582
Executive Committee of the World's Columbian Dental Congress—Work of the, by J. Taft, D.D.S. 55

PAGE

Exhibits at the World's Columbian Dental Congress 66
Experiments with Bichloride of Mercury, by Carrie M. Stewart, D.D.S. 8
Exostosis—Indications of Dental, by Mary Linde 269
Fractures of the Anterior Teeth—Conservative Method of Treatment, by Dr. Geo. W. Whitefield 13
Fillings—Anterior, by Dr. E. P. Beadles 478
Filling—Some of the Merits of a Contour Filling, by Dr. T. W. Foster 479
Harris Memorial, Chapin A... 548
Hygiene in Prosthetic Dentistry, by Dr. John A. Daly. 476
Hygiene—Report of, by Dr. W. E. Walker 538
Hygiene—Oral, by Dr. R. R. Freeman 539
Hypnotic Suggestion as a Dental Obtundent and Sedative, by Dr. Thos. Fillebrown 3
Hypnotism, by Prof. Geo. H. Mead 380
Infection from the Mouth, by L. E. Clifford, D.D.S. 194, 209
Inferior Maxilla—What Neoplasms Necessitate the Excision of the—Discussion. 11
Inter-Articulation — Some Facts Showing the Relationship of, by L. Van Orden, M.D. 7
Indigestion—Relation of Dental Caries to, by E. F. Wilson, M.D. 78
Ignorance and Negligence of Parents in the Care of Children's Teeth, by Dr. J. D. Adair 426
Jaws—A Study of the Masticating Force, by Dr. Geo. J. Dennis 26
Jaws and Teeth—Some of the Forces that Influence the Form of the, by Dr. W. X. Sudduth 59
Kalium—Natrium, Method of using, by Dr. J. D. Adair. 420

PAGE

Local Anæsthetics, by L. E. Custer, D.D.S.................. 527
Local Obtundents, by A. W. Haidle, D.D.S................ 338
Logan Crown—Method of Securing a Perfect Adaptation of, by Dr. T. P. Hinman............................... 419
Logan Crown—Simple Method of Banding, by Dr. T. P. Hinman......................... 470
Luxation or the Immediate Method in the Treatment of Irregular Teeth, by Dr. Geo. Cunningham......... 65
Masticating Force of the Jaws. by Dr. Geo. J. Dennis..... 26
Maxillary Bones and their Periosteum—Diseases of, by Vida A. Latham, D.D.S ........................157, 223, 261
Maxillary Sinus—Abscess of, by Dr. E. Lecaudey......... 9
Maxillary Sinus—Empyema of, by M. H. Fletcher, M.D., D.D.S.................. 105
Medicine—What Relation shall Dentistry Hold to—Discussion...................... 54
Medicine—Dentistry as a Specialty of, by Dr. R. A. Rush.............................. 432
Mouth—Infection from the, by E. L. Clifford, D.D.S. ............................194, 209
Nerve—Extraction of the Trigeminal by Dr. T. W. Brophy .............................. 582
Nitrous Oxide, by J. Lathrop, Jr., D.D.S..................... 320
Nomenclature—Correct, by Dr. Merrill..................... 424
Nomenclature—Report by Dr. S. H. Guilford............... 550
Oral Hygiene, by Dr. R. R. Freeman........................ 539
Oral Therapeutics—Practical, by Dr. Geo. V. Brown, M.D.................................. 172
Operative Technics, by Dr. D. M. Cattell..................... 15
Orthodontia—An Interesting Case in, by Dr. W. G. Browne......................... 422

PAGE

Obtundents—Local, by A. W. Haidle, D.D.S................ 338
Oxyphosphate of Copper, by Dr. W. V. B. Ames......... 485
Pathology—Case in by Dr. Gaskell ......................... 588
Plate Retainer, by Wm. H. Steele, D.D.S.................. 143
Planting Teeth by Dr. Wm. N. Morrison....................... 583
Platinum—A new Practical Way of Fusing, by L. E. Custer, D.D.S.................. 90
Persistent Vitality, by F. O. Jacobs, D.D.S................ 399
Pericementitis—Aconite and Iodine in, by W. I. Jones, D.D.S.......................... 191
Porcelain Teeth—An Original Method of Protecting the Tips, by Dr. Thos. P. Hinman.................................. 469
Porcelain Work, by J. H. Downie, D.D.S............... 532
Prosthetic Dentistry—Demonstrations...................... 494
Prosthetic Dentistry—Hygiene in, by Dr. John A. Daly.................................. 476
Prosthetic Dentistry—Two Useful Ideas in, by Dr. L. G. Noel ......................... 474
Prosthetics — Unprofessional, by Dr. G. N. Johnson...... 475
Prudence and Gutta-Percha, by Dr. Geo. V. I. Brown.. 23
Pulp-Canal—Some Changes that Take Place in and Around, by Dr. D. E. Caush ............................ 61
Pulps—Devitalization of, by C. M. Wright, D.D.S...... 82
Pulp—Devitalization of Dental, by A. W. Diack, D.D.S 218
Physiological Chemistry and Therapeutics, by Dr. O. C. Farish .......................... 424
Pyorrhœa Alveolaris — The Etiological Classification of, by Dr. M. L. Rhein.... 552
Reforms, by Sigel Roush, A.M., M.D., D.D.S.................. 187
Restoratives and Resuscitants, by N. S. Hoff, D.D.S....... 365

PAGE

Root Canals—Sulphuric Acid for opening, by J. R. Callahan, D.D.S.................. 73
Root Filling, by Harry Baldwin, L.D.S.................... 242
Rubber—Vulcanizing, by Dr. Geo. B. Snow.................. 599
Sensitive Dentine—Anæsthetizing, by W. H. Van Deman, D.D.S.................... 385
Soda—Tetraborate of, by Mr. Denis.......................... 10
Some Facts Showing the Relationship of the Inter-Articulation, by L. Van Orden, M.D......................... 7
Some of the Forces that Influence the Form of the Jaws and Teeth during the Process of Development, by Dr. W. X. Sudduth..... 59
Sulphuric Acid for Opening Root Canals, by J. R. Callahan, D.D.S.................. 73
Surgical Engine, The, by Dr. W. G. A. Bonwill........... 456
Syphilis, by W. H. Whitslar, M.D., D.D.S................. 521
Systemic Medication in Dental Practice, by Edgar Palmer, D.D.S.................... 116
Technics—Operative, by Dr. D. M. Cattell................ 15
Teeth—Appliance for Regulating, by J. F. Steele, D.D.S......................... 205
Teeth—Articulation of Artificial, by G. Molyneaux, D.D.S....................127, 273
Teeth—Care of Infants', by Dr. W. H. Morrison............. 487
Teeth of Children, by Howard Van Antwerp, D.D.S...... 92
Teeth—Enemies of the Human, by A. M. Benson, M.D., D.D.S.................. 164
Teeth—Etiology of the Irregularity and Crowding of the, by Dr. C. L. Boyd........................... 431
Teeth—Ignorance and Negligence of Parents in the Care of Children's, by Dr. J. D. Adair................. 426
Teeth—Luxation in the Treatment of Irregular, by Dr. Geo. Cunningham........... 65
Teeth—Planting, by Dr. Wm. N. Morrison.................. 583
Therapeutics of Aconite and Iodine in Pericementitis, by W. I. Jones, D.D.S...... 191
Therapeutics—Practical Oral, by Geo. V. Brown, M.D... 172
Therapeutics — Physiological, Chemistry and, by Dr. O. C. Farish...................... 424
Varnishing Cavities, by Dr. W. G. Browne.............. 487
"Vis Medicatrix Naturæ." by W. H. Whitslar, M.D., D.D.S.......................... 86
Vitality—Persistent, by F. O. Jacobs, D.D.S....... ........ 399
Vulcanizing Rubber, by Dr. Geo. B. Snow.................. 599
What Neoplasms Necessitate the Excision of the Inferior Maxilla—Discussion...... ................. 11
World's Columbian Dental Congress—Report of Treasurer...................... 493

### SOCIETY PROCEEDINGS.

Alabama Dental Association.. 417
American Dental Association ......................489, 550, 578
American Dental Protective Association—Report of... 549
Mississippi Board of Dental Examiners..................... 253
Mississippi Valley Dental Association..................... 206
National Association of Dental Faculties................. 506
National Association of Dental Examiners............... 564
Southern Dental Association .......................434, 469, 538
Woman's Dental Association of the United States........ 28
World's Columbian Dental Congress.....................1, 53
World's Columbian Dental Congress—Report of........ 493

SELECTIONS.

PAGE
Amyl Nitrite...................... 410
Anæsthesia—Semi-Centennial of the Discovery........... 607
A Curious Case.................... 409
Bacterial Pathology ............ 606
Bled to Death from Tooth Extraction........................ 605
Breath—Offensive Odor of...... 299
Bicycle Riding..................... 146
Biology—Origin and Meaning of the Term.................. 45
Bismuth in Solution............ 410
Blood Corpuscles—Origin of Red.............................. 609
Can Women Farm?.............. 304
Clark—Sir Andrew, A Reminiscence of...................... 300
Chemistry—Synthetical, Its Relation to the Materia Medica of the Future...... 293
Cleft Palate—Improved Operation, by Mr. Davies-Colley......................... 302
Cocaine Anæsthesia—Rendered Harmless.................. 606
Cocaine for Local Anæsthesia. 611
Cocaine—Use of.................. 43
Cocaine—In Surgical Operations—Indications for...... 408
Cocaine—Test...................... 222
Cold—How to avoid Taking... 38
Chloroform Anæsthesia without Danger.................... 516
Chloroform for External Hemorrhage................. 272
Chloroform Narcosis............ 607
Damages for Extracting a Sound Tooth................. 279
Dentistry in China............... 42
Dentition—Illnesses Ascribed to.............................. 241
Denture—Successful Removal from the Œsophagus...... 409
Diarrhœa of Dentition—Treatment.......................... 272
Disease—Prevention of.......... 305
Dunn Syringe..................... 537
Dyspepsia—Importance of..... 145
Every Man to His Trade...... 488
Food Hygiene..................... 201
Food Values—A Table of...... 147

PAGE
Glass Eyes—Price of........... 306
Gastric Intestinal Affections... 148
Healthiest Capital in the World........................... 518
Kimber—Diana Clifford........ 72
Lepers—A Hospital of......... 292
Longevity, by Dr. D. E. Nelson, M.D........................ 280
Longevity—Increasing......... 299
Medicine—The International Congress of..................... 292
Mind and Weather, The........ 199
Milk—Absorption of Odors by 609
Morphine Poisoning—Potassium Permanganate in..... 608
Œsophagus—Successful Removal of a Denture from the............................... 409
Patent Medicines.................. 609
Quack—Squelching a........... 144
Reminiscence of Sir Andrew Clark ........................... 300
Reynolds—Dr. Annie Felton.. 124
Right-Sightedness and Left-Sightedness..................... 356
Root Filling........................ 242
Sensitive Dentine—Obtundent 410
Spectacles—Superfluous......... 149
Scurvy in Children............... 297
Sugar—Value of and Effect of Smoking on Muscular Work........................... 515
Surgical Engine—The........... 456
Squelching a Quack............. 144
Synthetical Chemistry—Its Relation to the Materia Medica of the Future....... 293
Syphilis—Diagnosis of Hereditary............................. 43
Tait—Dr. Lawson........ ...146, 606
The Tongue in Disease.......... 604
Trional............................... 163
Title of Professor—The......... 518
Value of Sugar and Effect of Smoking on Muscular Work........................... 515
Voice—The Influence of Odors on the.......................... 304
What Caused Death............. 410
What Shall we Eat............... 459
Weather and the Mind—The.. 199
Woman's Health Protective Association.................... 149
Woman Honored—Another... 612

PAGE

Women as Sanitary Inspectors. 612
What Patients tell Experts—Rules Concerning.......... 612
Why are we Weary.............. 513

COLLEGE COMMENCEMENTS

American College of Dental Surgery........................ 230
Atlanta Dental College......... 239
Baltimore College of Dental Surgery........................ 232
Birmingham Dental College... 359
Boston Dental College.......... 462
Chicago College of Dental Surgery............................. 412
Cincinnati College of Dental Surgery........................ 235
Cleveland University of Medicine and Surgery, Dental Department.................. 237
Columbian University, Dental Department................. 361
Harvard University, Dental Department.................. 414
Indiana Dental College......... 232
Kansas City Dental College.... 236
Meharry Medical College, Dental Department............... 235
Missouri Dental College......... 239
National University, Dental Department.................. 358
New York College of Dentistry 233
Northwestern University Dental School..................... 412
Ohio College of Dental Surgery............................. 231
Ohio Medical University, Department of Dentistry.. 236
Pennsylvania College of Dental Surgery.................... 238
Philadelphia Dental College... 233
Royal College of Dental Surgeons of Ontario............ 230
Southern Medical College, Dental Department......... 359
University of Buffalo, Dental Department.................. 413
University of California, College of Dentistry............ 413
University of Denver, Dental Department.................. 240
University of Iowa, Dental Department.................. 237

PAGE

University of Maryland, Department of Dental Surgery.......................... 234
University of Michigan, College of Dental Surgery.... 360
University of Minnesota, Dental Department.............. 411
University of Pennsylvania, Department of Dentistry. 359
Vanderbilt University, Den- Department.................. 241
Western Dental College......... 240
Western Reserve University, Dental Department......... 236

EDITORIAL.

American Dental Association. ..........................310, 362
American Medical Association 102
An Interesting Case............. 47
An Inquiry......................... 47
Care of the Mouth and Teeth. 152
Degree—A New.................. 100
Dental College—A New......... 364
Dental Colleges—Opening...... 571
Dental Journal—A New........ 613
Dental Law of Ohio—Change in.............................. 309
Dentistry in Court................ 156
Editorial Retirement............ 103
Mississippi Valley Dental Society.......................50, 613
National Association of Dental Faculties.................. 363
National Meetings at Old Point Comfort....................... 465
Ohio State Dental Society, 49, 570
Professional Card—A........... 311
Professional Ethics.............. 363
Retirement........................ 103
Removal........................... 364
San Francisco Dental Society. 154
Shelley—Dr. Henry.............. 312
Southern Dental Association.. 364
Teeth—Care of the Mouth and 152
Tri-State Dental Meeting, 154, 520
Union.............................. 569
Wells' Memorial.................. 468

NOTICES.

Alabama Dental Association.. 151
American Dental Society of Europe.............362, 414, 569

PAGE

American Medical Association—Dental Section.......151, 250
Alumni Reunion................. 415
Chicago Dental Society......... 251
Connecticut State Dental Association........................ 416
East Tennessee Dental Association................ ............ 462
Illinois State Dental Society.........................................150, 308
Indiana State Dental Society.. 308
Iowa and Nebraska State Dental Societies—Joint Meeting............................... 203
Kentucky State Dental Association......................... 307
Midwinter Fair—Dental Congress......................... .. 251
National Association of Dental Faculties...................... 363
New England Dental Society.. 519
New York State Dental Society 252
Northern Ohio Dental Association............................ 203
San Francisco Dental Association............................ 203
Southern Dental Association... 364
Texas State Dental Association 253
World's Columbian Dental Congress—Transactions of 151

BIBLIOGRAPHICAL.

Care of the Mouth and Teeth—Popular Essays on, by Victor C. Bell, A.B., D.D.S 155
Catching's Compendium of Practical Dentistry for 1893......................... 311
Compend of Dental Prosthesis and Metallurgy, by Geo. W. Warren, D.D.S......... 614
Diseases and Injuries of the Teeth, by Morton Smale, M.R.C.S., L.D.S.........52, 101
Etiology of Osseous Deformities of the Head, Face, etc., by Eugene S. Talbot, M.D., D.D.S......................... 254
Practical Treatise on Mechanical Dentistry, by J. Richardson, M.D., D.D.S....... 51
Sanitarian, The, by Dr. A. N. Bell......................... 208
Traver's Dental Examination Register...................... 208

DENTAL LEGISLATION.

Dental Law of Arizona......... 35
Dental Law of Michigan........ 403
Dental Law of Ohio............. 309
Dental Law of Virginia......... 601

CORRESPONDENCE.

Letter from E. P. Cunningham, D.D.S......................... 204
Letter from Dr. J. W. Kasbeer 463
Mississippi State Board of Dental Examiners, by W. E. Walker, D.D.S.......... 253
Regulating Teeth, by J. F. Steele, D.D.S................. 205

IN MEMORIAM.

Dr. Samuel Wardle.............. 103

OBITUARY.

Wm. Henry Eames, D.D.S..... 259
Dr. Corydon L. Ford............. 255
Dr. A. O. Rawls................... 257
Dr. Henry Shelley................ 312
Dr. R. B. Winder................. 416

### DIRECTORY OF DENTAL SOCIETIES.

*American Dental Association*—Meets first Tuesday in August, 1894, at Old Point Comfort. Pres., J. D. Patterson, Kansas City, Mo.; Rec. Sec., Geo. H. Cushing, Chicago.

*Southern Dental Association*—Will meet in 1894. At Old Point Comfort. Pres., B. Holly Smith, Baltimore, Md.; Secretary, D. R. Stubblefield, Nashville, Tenn.

### STATE DENTAL SOCIETIES.

*Alabama Dental Association*—Meets annually. Next meeting at Troy, on the second Tuesday of April, 1894. Pres. T. M. Allen, Birmingham; Sec. S. W. Foster, Decatur.

*California State Dental Association*—Next meeting will be held in San Francisco, July, 1894. Pres., L. A. Teague, San Francisco; Rec. Sec., W. Z. King, San Francisco; Cor. Sec., C. E. Post, San Francisco.

*Colorado State Dental Society*—Meets 1st Tuesday in June, 1894, at Glenwood Springs. Pres. P. T. Smith, Denver; Sec., Sarah May Townsend, Denver.

*Connecticut State Dental Society*—Will hold a Union Meeting in connection with the Connecticut Valley Dental Society at Hartford, third Tuesday in May, 1894. Pres. C. C. Barker, Meriden; Rec. Sec. Geo. L. Parmele, Hartford.

*Dental Society of the State of Maryland and District of Columbia*—Meets annually. Next meeting at Washington City, on the second Tuesday in October, 1893. Pres. B. F. Coy, of Baltimore: Sec. M. W. Foster, Baltimore.

*Florida State Dental Association*—Meets annually. Next meeting at Talahassee, 1st Tuesday in May, 1894. Pres, F. E. Buck, Jacksonville; Cor. Sec. E. M. Sanderson, Jacksonville

*Georgia State Dental Society*—Meets in June, 1894, at Tybee Island; Pres. N. A. Williams, Valdosta; Rec. Sec. S. H. McKee, Americus; Cor. Sec. O. H. Mc Donald, Griffin.

*Illinois State Dental Society*—The next meeting will be held at Springfield, Ill., the second Tuesday in May, 1894. Pres. Garrett Newkirk, Chicago; Sec. Louis Ottofy, Chicago.

*Indiana State Dental Society*—Will meet at Lake Maxinkuckee, on the last Tuesday of June, 1894. Pres. W. M. Hindman, Vincennes; Secretary, G. E. Hunt Indianapolis.

*Iowa State Dental Society*—Meets annually. Next meeting in Council Bluffs, on the second Tuesday in May, 1894. Pres. S. E. Hatch, Sioux City; Rec. Sec. G. W. Miller, Des Moines.

*Kentucky State Dental Association*—Meets annually. Next meeting in Louisville, Ky., 3rd Tuesday in June, 1894. Pres., J. H. Letcher, Danville; Sec., J. H. Baldwin, Louisville.

*Kansas State Dental Association*—Pres. C. E. Esterly, Laurence; Sec., J. P. Root, Kansas City. Next meeting will be held at Topeka, last Tuesday in April, 1894.

*Maine Dental Society*—Will meet on the third Tuesday in July, 1894, in Rockland. Pres. H. A. Kelley, Portland; Sec. C. E. Bryant, Pittsfield.

*Maryland State Dental Association*—Next annual meeting will be held (place not definitely known yet,) in June, 1894. Pres., B. Holly Smith, Baltimore; Rec. Sec, W. W. Dunbracco, Baltimore; Cor. Sec., R. C. Bradshaw, Baltimore.

*Massachusetts Dental Society*—Meets annually in Boston, in June. Pres. Josiah W. Ball, Boston; Rec. Sec., E. O. Kinsman, Cambridge.

*Michigan State Dental Association*—Meets annually. Next meeting at Ann Arbor, Tuesday, June 7th, 1894. Pres., N. S. Hoff: Ann Arbor; Sec., J. Ward House, Grand Rapids. Treas. Geo. H. Mosher, Jackson.

*Mississippi State Dental Association*—Next meeting the first Tuesday in May, 1894, at Natchez, Miss. Pres. Jas. B. Askew, Vicksburg; Cor. Sec. J. D. Killian, Greenville.

*Minnesota State Society*—Pres. C. H. Stearns, Zumbrota; Sec, C. W. Jones, St. Paul. Will meet in Minneapolis, first week, in September, 1894.

*Missouri Dental Association*—Meets at Excelsior Springs, Mo., second Tuesday in July, 1894. Pres., W. E. Tucker, Springfield, Mo.; Sec., Wm. Conrad, St. Louis.

*Nebraska State Dental Society*—Meets annually. Next meeting third Tuesday in May 1893, at Beatrice; Pres. J. J. Willey, Wahoo; Sec. C. R. Tefft, Lincoln.

*New Jersey State Dental Society*—Meets annually. The next meeting will be held at Asbury Park, onthe 18th, 19th, 20th, of July, 1894. Pres. E. M. Beesley, Belvidere; Sec. C. A. Meeker, Newark.

*New Hampshire Dental Society*—Meets Annually. Next meeting will be held in Concord, N. H.; time to be decided later. Pres. W. R. Blackstone, Manchester; Sec. Burton C. Russell, Keene.

*North Carolina State Dental Society*—Meets in Durham, on the first Tuesday in May, 1894. Pres C. A. Rominger, Reidsville; Sec., J. E. Wyche, Oxford.

*North Dakota State Dental Society*—The next meeting will be held in Grand Forks, in 1894, the exact time will be given later. Pres. B. D. McLain, of Jamestown; Sec. R. B. Foster, Grand Forks.

*Ohio State Dental Society*—Meets annually. Next meeting at Columbus, first Tuesday of December, 1894. Pres. Chas. Welch, Wilmington; Sec. L. P. Bethel, Kent.

*Pennsylvania State Dental Society*—Will hold its next annual meeting at Cresson, Pennsylvania, July 11th, 1893. Pres., W. E. VanOrsdel, Sharon; Cor. Sec., C. V. Kratzer, Reading, Pa.

*Rhode Island Dental Association*—Pres., S. W. Clark, Providence; Sec., H. W. Gillett, Newport, Meets quarterly, second Tuesday in July, October, January and April. Annual Meeting second Tuesday in July, at Providence.

*South Carolina State Dental Association*—Meets annually. Pres. C. S. Patrick, Charleston; Cor. Sec. H. J. Ray, Aiken. Next meeting at Columbia, S. C., in August, 1893.

*Tennessee Dental Association*—Meets annually. Next meeting at Nashville, first Tuesday in July, 1894. Pres. W. J. Morrison, Nashville; Rec Sec. W. W. Jones, Murfreesboro; Cor. Sec. S B. Cook, Chattanooga.

*The Dental Society of the State of New York*—Meets annually on the second Wednesday in May. Next session at Albany, 9th and 10th of May, 1894. Pres. F. T. Van Woert, Brooklyn; Sec. C. S. Butler, Buffalo; Cor. Sec. R. Ottolengui, New York.

*Vermont State Dental Society*—Meets annually. Next meeting at White River Junction, Vt., third Wednesday in March, 1894. Pres. A. J. Parker, Bellows Falls; Sec. Thos. Mound, Rutland.

*Virginia State Dental Association*—Will meet at Old Point Comfort, in 1894, Exact tine to be decided later. Pres. H. W. Campbell, Suffolk; Sec. J. Hall Moore, Richmond.

*Washington State Dental Society*—Meets Annually. Next meeting will be held at Tacoma, in the early part of May, 1894. Pres. P. H. Carlyon, Olympia; Sec., A. S. Oliver, Olympia.

*Wisconsin State Dental Society*—Meets annually. Next meeting at will be held at Janesville, Wis., the second Tuesday in July, 1894. Pres., W. C. Wendel, Milwaukee; Sec., C. A. Southwell, Milwaukee.

*West Virginia State Society.*—The annual meeting will be held at Parkersburg, on the first Wednesday of October, 1893. Pres. J. N. Mahan, Charleston; Sec. Geo. I. Keener, Morgantown.

### LOCAL SOCIETIES.

*American Academy of Dental Science*—Meets annually in Boston, Mass., on the first Wednesday in October. Pres. J. H. Batchelder; Rec. Sec. E. E. Hopkins; Cor. Sec. E. B. Hitchcock, Boston.

*Buffalo Dental Association*—Meets last Monday evening of each month. Annual meeting in June. Pres. F. H. Boswell; Sec. J. A. Stackhouse.

*Brooklyn Dental Society and Second District Society United*—Meets quarterly—Pres. T. H. Holly; Rec. Sec. A. N. Russell, Brooklyn.

*Central Dental Association of Northern New Jersey*—Meets bi-monthly; and annually. The next annual meeting will be held about the middle of February, 1894. Pres. Harvey Iredell, New Brunswick: Sec. Wm. L. Fish, Newark.

*Chicago Dental Society*—Meets monthly. Pres. J. W. Wassall; Sec. L. L. Davis, Cor. Sec. Geo. J. Dennis.

*Chicago Dental Club*—Meets on the fourth Monday of each month. Pres. A. B. Freeman; Sec. C. Stoddard Smith.

*Cleveland Dental Society.*—Meets the first Monday of each month. Pres. S. B. Dewey; Sec. Martha J. Robinson.

*Connecticut Valley Dental Society*—Meets annually. Next meeting at Hartford, May, 1894. Pres. H. Rider, Danbury, Mass.; Sec. Geo. A. Maxfield, Holyoke, Mass.

*Detroit Dental Society*—Meets monthly, Pres. E. C. Moore, Sec. H. C. Raymond.

*Eighth District Dental Society, N. Y.*—Next annual meeting April 24th, 1894, in Buffalo. Pres. F. J. Burkhart, Batavia; Sec., W. V. Grove, Buffalo

*East Tennessee Dental Association*—Meets annually. Next meeting will be held at Athens, Tenn., in August, 1893. Pres. U. D. Billmeyer, Chattanooga; Sec. and Treas. A. W. Palmer, Chattanooga.

*Fifth District Dental Society of N. Y.*—Meets semi-annually. Next meeting in Syracuse, October, 24-26. Pres. R. F. Jones, Utica; Sec. T. B. Mason, Phoenix.

*First District Dental Society of the State of New York*—Meets annually. The next meeting will be held on the second Tuesday of October, 1892. Pres., Wm. Carr, New York City; Sec., Benj. C. Nash, New York City.

*First District Dental Society of Illinois*—Next meeting will be held in Peoria, second Tuesday in September, 1894. Pres. O. M. Daymunde, of Monmouth; Sec. W. O. Butter, La Harpe.

*Knoxville City Dental Society*—Meets monthly. President, J. T. Cazier; Sec. A. J. Cottrell.

*Mississippi Valley Dental Society*—Meets annually at Cincinnati, next Meeting March, 1894. Pres., O. N. Heise, Cincinnati; Sec. H. T. Smith, Cincinnati; Cor. Sec. H. C. Matlack Cincinnati, O.

*New England Dental Society*—Meets annually. Next meeting first Thursday in Oct., 1892, in Boston Mass. Pres. J. F. Adams, Worcester, N. H.; Sec. E. O. Kinsman, Cambridge, Mass.

*Northern Illinois Dental Society*—Meets annually. Next meeting at Aurora, in October, 1894. Pres. E. R. Warner, Chicago; Sec. J. W. Cormany, Mt. Carroll.

*Northern Ohio Dental Association*—Meets annually. Next meeting at Put-in-Bay, O., on the 2d Tuesday in May, 1894. Pres. S. B. Dewey, Cleveland; Rec. Sec. F. H. Knowlton, Akron; Cor. Sec. J. F. Dougherty, Canton.

*Northwestern Dental Association* (embraces portions of Dakota, Minnesota and Manitoba) meets annually. Next meeting at Detroit, Dak. on the fourth Tuesday in July, 1892. Pres. J. W. Cloes, Jamestown, Dak. Sec. S. J. Hill, Fargo, Dak.

*Odontological Society of Pennsylvania*—Meets on the first Saturday evening of each month, at 8 o'clock p. m., at 13th and Arch sts; Pres. Jas. Truman, Rec. Sec. A. W. Deane.

*Odontological Society of New York City*—Meets the third Tuesday of each month. Pres. Wm. Jarvie; Rec. Sec. E. T. Payne.

*Odontological Society of Cincinnati*—Meets monthly. Pres. G. Molyneaux; Sec. H. C. Matlack.

*Odontological Society of Chicago*—Meets monthly. Pres. W. V. B. Ames; Sec., and Treas., Louis Ottofy.

*Pennsylvania Association of Dental Surgeons*—Meets second Tuesday of each month. Pres. H. E. Roberts; Cor. Sec. Theo. F. Chupein.

*Pittsburg Dental Society*—Meets the last Tuesday Evening of each month. Pres. J. B. Williams, Homestead; Sec. N. L. Reinecke.

*San Francisco Dental Society*—Meets the second Monday evening of each month. Pres. W. A. Knowles; Sec. Chas. Morffew; Treas. J. J. Birge.

*Sixth District Dental Society of N. Y.*—Meets semi-annually. The annual meeting will be held the first week in May, 1893. Pres., Chas. W. McCall, Binghamton; Sec., T. B. Fuller, Binghamton.

*Seventh District Dental Society of Ohio*—Meets annually. Next meeting in May, 1894. Pres., O. N. Heise, Cincinnati; Sec., J. R. Callahan, Cincinnati.

*Southern Illinois Dental Society* Meets annually. Next annual meeting will be held at Nashville, Ill., on the 3d Tuesday in October, 1894. Pres., L. B. Torrence, Chester; Sec., G. W. Entsminger, Carbondale.

*Southern Minnesota Dental Society*—Next meeting in Mankato, second Tuesday in April, 1894. Pres. M. B. Wood, Mankato; Sec. F. B. Kremer, Minneapolis.

*St. Louis Dental Society*—Meets first and third Tuesday in each month. Pres. Dr. DeCourcy Lindsley; Cor. Sec. Wm. Conrad; Rec. Sec. J. G. Pfaff.

*The Isaac Knapp Dental Coterie of Fort Wayne, Ind.*—Meets first and third Tuesday in each month. Pres., H. C. Sites; Sec., J. S. McCurdy.

*The Minneapolis Dental Society Monthly*—Pres. E. F. Clark, Sec. E. J. Morrison.

*The Tacoma Dental Society*—Meets monthly. Pres. M. C. Burns; Sec. W. E. Burkhart.

*Washington City Dental Society*—Meets 3d Tuesday of each month. Pres. J. H. P. Benson; Rec. Sec. J. Roland Walton.

### Mississippi Valley Dental Society.

The annual meeting of this body will be held in Cincinnati early in March next, and we would simply suggest that the officers give the matter attention at as early a day as possible, for this should be a grand, good meeting. Being the fiftieth year of the existence of the society, it should have a semi-centennial meeting that should command the attention of its entire membership and also of the profession outside.

It is to be hoped that the Executive Committee will act promptly, and secure an attractive and interesting programme for that occasion. The Executive Committee is distributed throughout the various neighboring States, and we would suggest that their attention be at once given to this matter, and that they make such preparation as shall result in a meeting worthy of the occasion and worthy of this pioneer society.

The REGISTER will publish any suggestions or plans in reference to the matter from the officers or the Executive Committee.

It is to be hoped that a definite time will be selected that will be most favorable for bringing together the largest possible number; and let all come with a full determination to make this meeting an eminent success, and to give it the entire attention during the time of the meeting.

The Ohio State Dental Society.............................. 49

Mississippi Valley Dental Society.............................. 50

Bibliographical.............................. 51

---

### THE DENTAL REGISTER,

A MONTHLY MAGAZINE DEVOTED TO THE INTERESTS OF THE

**DENTAL PROFESSION.**

*Terms Reduced to* $2.00 *Per Year. Single Copies,* 25 *Cents.*

**EDITED BY PROF. J. TAFT, D.D.S.**

—AND—

**Published by SAM'L A. CROCKER & CO., Ohio Dental and Surgical Depot.**

The volume commences in January and closes in December.

The REGISTER being issued monthly is always fresh, therefore Indispensable to the practitioner who desires to keep posted up with the new inventions and improvements which are daily developed. Neither expense nor effort will be spared to give value and variety to its contents. Articles will be illustrated with well executed engravings whenever they will add to the interest or assist to explanation.

Its extensive circulation and frequency of issue make it one of the BEST DENTAL ADVERTISING MEDIUMS in the United States.

All subscriptions must begin with January or July.

Local or special notices, five lines or less, will be inserted for $3 each insertion; over five lines, 50 cts. per line.

All advertising bills payable in advance, or at furthest, after the issue of the first number containing the advertisement. All transient advertisements to be paid for in advance.

☞CONTRIBUTIONS SOLICITED.

Exclusively Editorial Communications should be addressed to the Editor.

The latest number of the REGISTER may be ordered with or without other goods at any time, and all letters should be addressed to

